# Integrated ‘all-in-one’ strategy to stabilize zinc anodes for high-performance zinc-ion batteries

**DOI:** 10.1093/nsr/nwab177

**Published:** 2021-09-15

**Authors:** Canpeng Li, Xuesong Xie, Hui Liu, Pinji Wang, Canbin Deng, Bingan Lu, Jiang Zhou, Shuquan Liang

**Affiliations:** School of Materials Science and Engineering, Key Laboratory of Electronic Packaging and Advanced Functional Materials of Hunan Province, Central South University, Changsha 410083, China; School of Materials Science and Engineering, Key Laboratory of Electronic Packaging and Advanced Functional Materials of Hunan Province, Central South University, Changsha 410083, China; School of Materials Science and Engineering, Key Laboratory of Electronic Packaging and Advanced Functional Materials of Hunan Province, Central South University, Changsha 410083, China; School of Materials Science and Engineering, Key Laboratory of Electronic Packaging and Advanced Functional Materials of Hunan Province, Central South University, Changsha 410083, China; School of Materials Science and Engineering, Key Laboratory of Electronic Packaging and Advanced Functional Materials of Hunan Province, Central South University, Changsha 410083, China; School of Physics and Electronics, Hunan University, Changsha 410082, China; School of Materials Science and Engineering, Key Laboratory of Electronic Packaging and Advanced Functional Materials of Hunan Province, Central South University, Changsha 410083, China; School of Materials Science and Engineering, Key Laboratory of Electronic Packaging and Advanced Functional Materials of Hunan Province, Central South University, Changsha 410083, China

**Keywords:** all-in-one electrode, hydrogen-free, electrode/electrolyte interface, Zn-based batteries

## Abstract

Many optimization strategies have been employed to stabilize zinc anodes of zinc-ion batteries (ZIBs). Although these commonly used strategies can improve anode performance, they simultaneously induce specific issues. In this study, through the combination of structural design, interface modification, and electrolyte optimization, an ‘all-in-one’ (AIO) electrode was developed. Compared to the three-dimensional (3D) anode in routine liquid electrolytes, the new AIO electrode can greatly suppress gas evolution and the occurrence of side reactions induced by active water molecules, while retaining the merits of a 3D anode. Moreover, the integrated AIO strategy achieves a sufficient electrode/electrolyte interface contact area, so that the electrode can promote electron/ion transfer, and ensure a fast and complete redox reaction. As a result, it achieves excellent shelving-restoring ability (60 hours, four times) and 1200 cycles of long-term stability without apparent polarization. When paired with two common cathode materials used in ZIBs (*α*-MnO_2_ and NH_4_V_4_O_10_), full batteries with the AIO electrode demonstrate high capacity and good stability. The strategy of the ‘all-in-one’ architectural design is enlightened to solve the issues of zinc anodes in advanced Zn-based batteries.

## Teaser

The integrated ‘all-in-one’ electrode combines the merits of structural design, interface modification and electrolyte optimization, which can effectively promote the reversibility and stability of zinc-based batteries.

## INTRODUCTION

Aqueous zinc-ion batteries (ZIBs), with their cost efficiency, high safety, nontoxic features and high energy density, are quite competitive and popular in the large-scale energy storage and wearable electronics field [1–3]. Since the reversible zinc-ion storage in aqueous system, numerous breakthroughs have been made in research into cathode materials [4–9]. Commercial zinc foil has been used in anode materials, but little has been done to overcome its inherent problems [10]. In the past two years, the use of zinc metal anodes has been attracting more attention, with several studies summarizing issues and proposed relevant optimizations [11–13]. Recent reviews on the anodes of ZIBs described the main issues as formation of zinc dendrites, hydrogen evolution, corrosion and passivation [14,15]. The current modification strategies include structural design [16,17], surface modification [18], electrolyte optimization [19] and zinc alloying [20]. Structural design is a widely employed method of modification. The essence of this method is to increase the specific surface area of the electrode to accelerate distribution of the electrolyte and current uniformly on the electrode surface, thereby achieving uniform deposition of zinc ions [21]. Therefore, more attention should be directed toward the research progress of three-dimensional (3D) zinc anode than non-3D anodes [22]. Despite these advantages, however, traditional 3D anodes employed in routine liquid electrolytes show an increase in the specific surface area, which, in turn, indicates a reduction in the local current density. According to the Tafel formula, hydrogen evolution overpotential should decrease. In addition, as the specific surface area increases, there will inevitably be more reactive sites on the anode surface. With this comes the probability of an increase in hydrogen evolution and other side reactions. The increase in these reactions will greatly reduce the Coulombic efficiency (CE) of zinc deposition/stripping, and thus, the cycle life of the zinc anode, thereby affecting the cycle performance of the battery.

Interface modification is a commonly used strategy to reduce side reactions caused by active water molecules [23]. This strategy avoids direct contact between the electrolyte and electrode. Most interface modification strategies can realize uniform zinc deposition, selective ion transfer and anti-corrosion properties [24,25]. However, the introduced coating layer increases the internal impedance and hinders the rapid transport of ions and electrons.

Flexible batteries are a promising developmental direction for ZIBs [26,27], and gel electrolytes account for a large proportion of the electrolytes they employ [28,29]. However, because of differences in electrolyte fluidity, their development is significantly restricted by electrode/electrolyte interface issues [30]. This is because of limited contact area, volume change and morphology change of the electrode during cycling. A solution to these problems is required to achieve sufficient and close contact between the electrolyte and electrode. For the above three anode modification strategies, an optimized approach is urgently required to combine their strengths.

**Figure 1.**
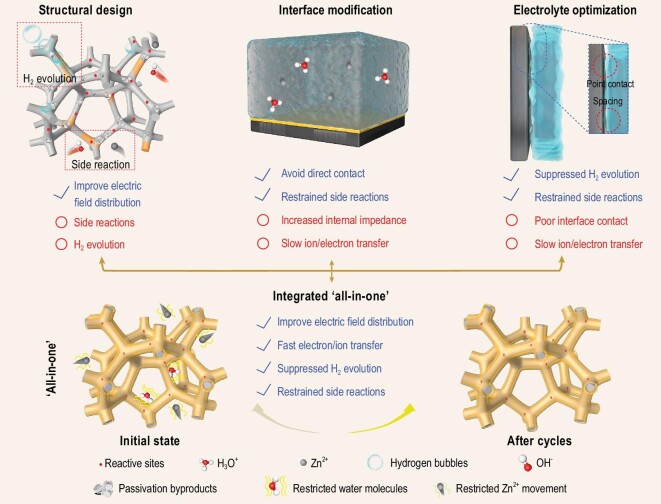
Schematics of structural design, interface modification, electrolyte optimization and integrated ‘all-in-one’ system, advantages and disadvantages are also listed.

Therefore, we designed an ‘all-in-one’ (AIO) electrode by combining the strategies of structural design (3D skeleton), interface modification (sufficient interface contact) and electrolyte optimization (mixed gel electrolyte). This integrated AIO strategy should increase the electrode/gel electrolyte contact area, facilitate occurrence of the ion transportation and redox reactions, and improve the adaptability of electrode volume changes to alleviate the interface stress problem. Additionally, the AIO electrode retains the advantages of a high specific surface area while effectively suppressing hydrogen evolution and side reactions, consequently achieving better stability (Fig. 1). In a symmetrical battery, the as-prepared AIO electrode achieved a 600-hour long-time stability without significant polarization, as well as ultra-stable shelving-restoring ability. In Zn/*α*-MnO_2_ and Zn/NH_4_V_4_O_10_ cells, the AIO electrode also exhibited better electrochemical performance than traditional 3D anodes in routine liquid electrolytes.

## RESULTS AND DISCUSSION

**Figure 2.**
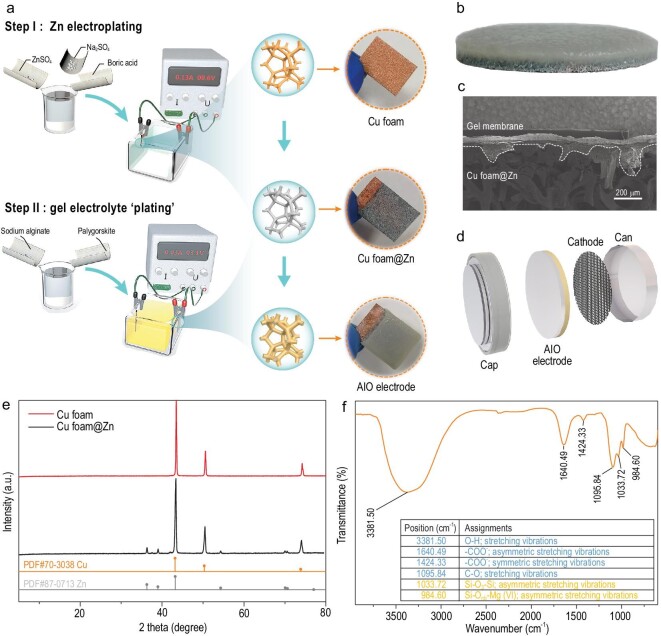
(a) Schematics of the two-step electroplating process for preparing AIO electrodes, and photos of Cu foam, Cu foam@Zn, and AIO electrodes. (b) Cross-sectional photo and (c) cross-sectional SEM image of the AIO electrode. (d) Diagram of battery assembly. (e) XRD patterns of Cu foam and Cu foam@Zn. (f) FT-IR spectra of gel membrane and its corresponding vibration form.

The preparation of the AIO electrode is shown in Fig. 2a. In step I, Cu foam and a Zn sheet are used as the working and counter electrodes, respectively, for the Zn plating to obtain Cu foam@Zn [31]. Using sodium alginate as the main content and palygorskite powder as an additive, a mixed electrolyte ‘plating’ suspension is obtained in step II. Subsequently, the Cu foam@Zn and a Zn sheet are utilized as the working electrode and counter electrodes, respectively, such that the Zn on Cu foam@Zn loses electrons and transfers into an electrolyte ‘plating’ suspension. Sodium alginate in the electroplating suspension completes the ionic cross-linking on the surface of the Cu foam@Zn. This is seen in Fourier transform infrared spectra (FT-IR) from the asymmetric stretching vibrations of the −COO^–^ groups, which shift from 1615 cm^–1^ to higher values 1640 cm^–1^ through formation of coordinate bonds between the carboxylate groups and zinc ions (Fig. S1, Supplementary data) [32,33], and brings the palygorskite together with the electrode to form an AIO electrode. Palygorskite has been proven to effectively improve battery performance through ion exchange [34]. Surface and cross-sectional energy dispersive X-ray (EDS) mappings (Al, Zn, Si, Mg) confirm the uniform distribution of the palygorskite material [MgAlSi_4_O_10_(OH)·4H_2_O] at the surface and body of the gel membrane (Fig. S2). Photographs of the Cu foam, Cu foam@Zn and AIO electrodes are shown in Fig. 2a. The distributions of Zn on the Cu foam and mixed gel membrane on the Cu foam@Zn are both quite uniform. The cross-sectional photograph and scanning electron microscope (SEM) image of the AIO electrode show that the gel membrane penetrated the electrode and was tightly bonded to Cu foam@Zn (Fig. 2b and c), achieving sufficient electrode/electrolyte interface contact. Such an AIO electrode can function as an anode, electrolyte and separator simultaneously, as shown in Fig. 2d. The X-ray diffraction (XRD) results show that Cu foam@Zn was obtained during the electroplating process without formation of by-products (Fig. 2e). Analysis of the peeled off electrolyte membrane by FT-IR shows that its infrared peaks (Fig. 2f) matched well with its two components—palygorskite and zinc alginate (Fig. S3a).

**Figure 3.**
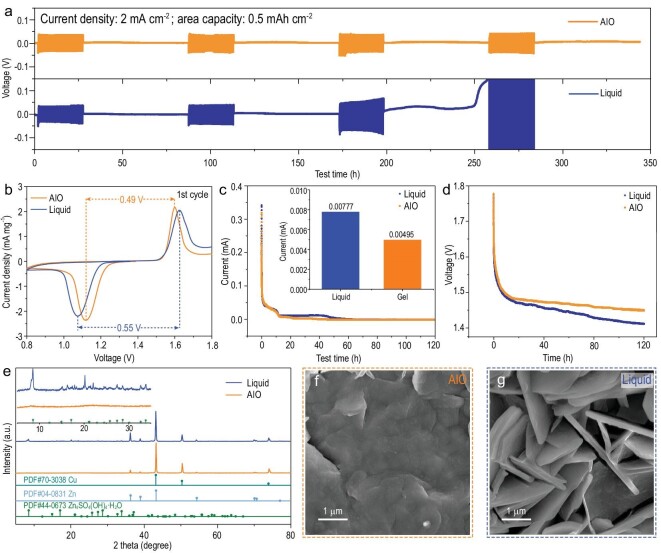
(a) Shelving-recovery performance of Cu foam@Zn/Cu foam@Zn symmetric cell (AIO electrode/Cu foam@Zn in AIO system) under 2 mA cm^–2^. (b) First cyclic voltammetry curve, (c) float charge current, (d) open circuit potential decays of AIO electrode/*α*-MnO_2_ and Cu foam@Zn/*α*-MnO_2_ full cell. (e) XRD patterns of the anodes in different full cell systems after 100 cycles at 500 mA g^–1^, and the corresponding SEM images of (f) AIO electrode and (g) Cu foam@Zn in 2 M ZnSO_4_ + 0.1 M MnSO_4_.

To illustrate the feasibility of this optimization strategy, the electrochemical performances of the AIO electrode and the Cu foam@Zn in liquid electrolytes (2 M ZnSO_4_ or 2 M ZnSO_4_ + 0.1 M MnSO_4_) were compared. In terms of shelving-recovery performance, the AIO system delivered a small polarization voltage after undergoing 60 hours of shelving thrice and maintained a normal open-circuit voltage (0.006 V) during the fourth shelving (Fig. 3a). In contrast, in the liquid system, after going through 60 hours of shelving twice, a significant polarization increment occurred in the third cycle. During the third shelving, the open circuit voltage increased sharply, accompanied by battery failure. In a symmetric battery, the AIO system exhibited better reversibility and stability. Cyclic voltammogram tests of a full battery (Cu foam@Zn/*α*-MnO_2_) show that the ΔV between the oxidation and reduction peaks in the AIO system is smaller than that in the liquid system (2 M ZnSO_4_ + 0.1 M MnSO_4_). This is observed in both the first (Fig. 3b) and second circles (Fig. S3b), indicating that the AIO system exhibits better reversibility.

Floating charge current can be used to evaluate the amount of energy required to maintain a battery at 100% charge. Generally, the smaller the floating charge current, the better the stability of the system [35,36]. As shown in Fig. 3c, the floating charge current of the liquid system was 0.00777 mA, whereas that of the AIO system was reduced by 36.3%. In addition, the AIO system effectively suppressed self-discharge (Fig. 3d). The faradaic reaction, which includes decomposition of the electrolyte, is the main cause of self-discharge. This suggests that the AIO system inhibits unwanted side reactions [37,38]. Zn_4_SO_4_(OH)_4_·*x*H_2_O is a common by-product in aqueous ZIBs, and its formation can be indicative of the severity of the side reactions. A comparison of the XRD patterns of the anode in the AIO system and liquid systems after 100 cycles at a current density of 500 mA g^–1^ shows that the AIO system can effectively inhibit formation of Zn_4_SO_4_(OH)_4_·*x*H_2_O (Fig. 3e). This conclusion can be verified by the SEM images of the anode in the AIO (Fig. 3f) and liquid (Fig. 3g) systems, and further confirmed by low magnification SEM images (Fig. S4). Moreover, the morphology of the anode after cycling in the AIO system is much flatter than that in the liquid system.

**Figure 4.**
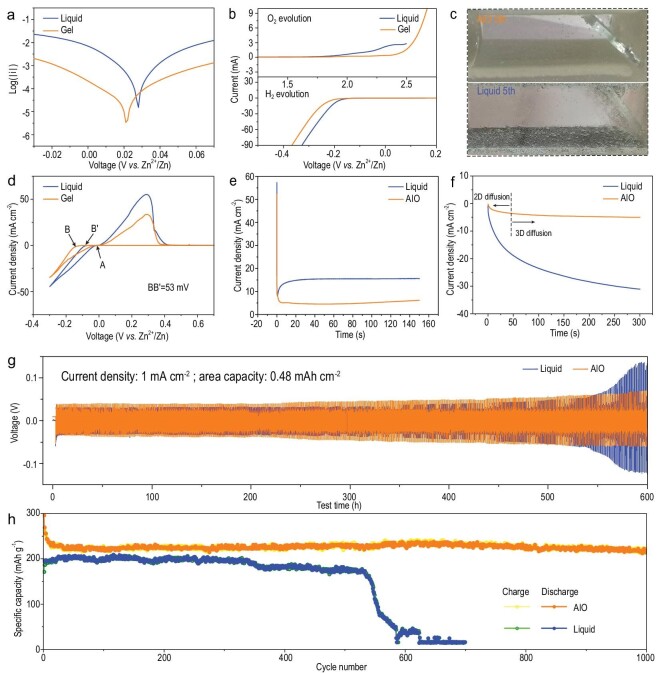
(a) Linear polarization curves of Zn foil/Zn foil symmetric cell in different electrolyte systems (liquid and gel, where the gel represents the electrolyte peeled from the electrode) at a scan rate of 5 mV s^–1^. (b) Linear sweep voltammetry curves of Zn foil/Ti foil cell at a scan rate of 0.05 V s^–1^. (c) Symmetrical cells with AIO electrode and Cu foam@Zn assembled in transparent tanks representing the side reactions visually during continuous Zn plating/stripping at 0.5 mA cm^–2^. (d) Cyclic voltammograms for Zn nucleation in AIO and liquid systems. (e) Chronoamperograms of AIO electrode/Cu foil and Cu foam@Zn/Cu foil cells at the same overpotential. (f) Chronoamperograms of AIO electrode/Zn foil and Cu foam@Zn/Zn foil cells. (g) Long-term galvanostatic cycling performance of symmetrical cells with AIO electrode and Cu foam@Zn at 1 mA cm^–2^. (h) Cycling performance of AIO electrode/NVO and Cu foam@Zn/NVO full cell at 10 A g^–1^.

To explore the reasons for the improved stability and reversibility of the AIO electrode, a linear polarization test was performed to compare the corrosion properties of Zn metal in the different systems. As shown in Fig. 4a, there was a smaller corrosion current in the gel system. It is generally believed that a lower corrosion current indicates a lower corrosion rate [39], which means that Zn metal exhibits greater stability in the AIO system. Comparing the stable electrochemical windows of the gel electrolyte and the liquid electrolyte, the former has both a higher O_2_ evolution potential and a lower H_2_ evolution potential (Fig. 4b). Thus, the AIO system can effectively relieve gas evolution [40]. To observe the hydrogen evolution more intuitively, symmetrical batteries were assembled in a transparent container to perform repeated Zn deposition/stripping. As shown in Fig. 4c, after five cycles under the same conditions (Fig. S3c), obvious bubbles can be observed on the electrode surface in the liquid system, whereas the formation of bubbles can barely be seen in the AIO system. With respect to the nucleation overpotential (NOP, Fig. 4d), that of the AIO system is 53 mV larger than that of the liquid system, which is mainly attributed to the interaction between zinc ions and carboxyl groups [33]. The larger NOP value provides a sufficient nucleation driving force to form finer nuclei [23]. Current change with time under a constant potential has the sensitivity to reflect the nucleation process and surface changes [41]. To analyze the deposition forms of zinc ions on copper and zinc in the two systems, the chronoamperometry comparisons of the Cu foam@Zn/Cu and Cu foam@Zn/Zn batteries were conducted. It can be seen from Fig. 4e that Zn deposition on the copper substrate in the AIO system is closely arranged. In the chronoamperometry of the Cu foam@Zn/Zn battery, two-dimensional diffusion corresponds to the unrestricted diffusion of zinc ions on the anode surface. The AIO system only undergoes a fast constrained 2D diffusion before entering a stable 3D diffusion stage (Fig. 4f), that is, zinc ions tend to be reduced at the adsorbed position, which greatly increases the number of nucleation points and improves the distribution situation. This limited 2D diffusion can also be attributed to the coordination between carboxyl groups and zinc ions [33].

The reasons for the improved stability of the AIO system are shown in Fig. 1. In detail, the increase in surface area through this structural design causes the local current density to diminish, and consequently, the hydrogen evolution overpotential to decrease [42]. In most 3D systems, the number of reactive sites inevitably increases because of surface area enlargement. Hence, H_3_O^+^ is more likely to obtain electrons to generate hydrogen (with the generated bubbles adhering to the electrode surface), which hinders the migration path of Zn^2+^. As hydrogen evolution and Zn^2+^ deposition are competitive reactions, the easier reduction of H_3_O^+^ means that it is more difficult for Zn^2+^ to obtain electrons [43]; that is, the zinc stripped from the Cu foam@Zn is more difficult to return. The macroscopic phenomenon is that zinc on Cu foam@Zn gradually dissolves as the cycle number increases. Moreover, the occurrence of hydrogen evolution means that the partially remaining OH^–^ aggravates side reactions and generates by-products such as Zn_4_SO_4_(OH)_4_·*x*H_2_O. For interface modification, introducing a coating layer usually leads to an increase in internal impedance, which hinders fast ion/electron transfer. Because most of the water molecules are fixed inside the gel, electrolyte optimization can effectively reduce the water-induced side reactions, but the poor interface contact blocks its further development. In contrast, within the AIO electrode, the advantages are cleverly combined. Most of the water molecules are fixed inside the mixed gel, and the number of active water molecules are markedly reduced. As a result, side reactions, such as hydrogen evolution caused by active water molecules, are greatly inhibited. Meanwhile, the role of the 3D structure in homogenizing ion deposition is retained, as the interaction between the gel electrolyte and Zn^2+^ has been strengthened to a certain extent. Moreover, the close contact between the gel membrane and the electrode can enable fast electron/ion transportation [44].

Because the AIO system is more stable than the liquid system, it is believed that batteries with an AIO electrode should also exhibit a better electrochemical performance. In 70 cycles of a Zn/Cu battery under a 20% depth of discharge (Fig. S5), the CE of the AIO system was maintained at ∼100%, while the liquid system had a relatively obvious voltage fluctuation at 41 cycles, and then completely failed in the 68th cycle (CE dropped to 0%). Because of the lack of a rigid substrate, the zinc foil completely failed after only 14 cycles under a 20% depth of discharge. In the Zn/Zn symmetric battery, the polarization voltage of the AIO system can be stabilized within ±0.06 V after cycling for 600 hours at a 7% depth of discharge, whereas the polarization voltage of the liquid system more than doubles (Fig. 4g). In addition, when the battery was disassembled to compare the electrodes (Fig. S6), the zinc on the Cu foam@Zn in the AIO system was still visible, whereas the zinc dissolution could be clearly observed in the liquid system, which is essentially a result of the reduction in CE caused by the severe hydrogen evolution mentioned above. Surface and cross-sectional SEM images (Fig. S7) of the AIO electrode after cycling also confirmed that the gel electrolyte was still coated onto the 3D structural anode, which is the same as the gel electrolyte before cycling with an obvious layer configuration. By comparing the stability of symmetrical batteries in the two systems at different current densities, it was found that the AIO system also shows a better rate performance (Fig. S8). At a current density of 4 mA cm^–2^, the polarization voltage of the liquid system increased sharply, followed by system failure, whereas the AIO system maintained a stable performance. The difference in rate performance can be attributed to the stability difference of the two systems, as well as the higher ionic conductivity of the AIO system (Fig. S9a). The high ionic conductivity may be associated with the abundant ion transfer channels of the nano-palygorskite materials [34]. As the electrochemical reaction is a coordinated process of electron transmission and ion migration, under a large current density, the ion migration is limited by the polarization of concentration; that is, systems with higher ionic conductivity tend to obtain better high-rate performance. Notably, the contact area provided by the close proximity of the gel membrane and electrode enables the high ionic conductivity of the gel electrolyte to be effectively utilized. Moreover, in terms of electron transmission, the AIO system possesses a smaller electric charge resistance, according to the Nyquist plots (Fig. S9b and c). In summary, fast ion/electron transfer can be realized using an AIO electrode.

The superiority of the rate performance is also reflected in the Cu foam@Zn/NH_4_V_4_O_10_ (NVO) (Fig. S9d) and Cu foam@Zn/*α*-MnO_2_ (Fig. S9e) full cells. Compared to the cycle performance of the Cu foam@Zn/*α*-MnO_2_ system at a current density of 0.5 A g^–1^ (Fig. S9f), the specific discharge capacities of the two 3D systems were similar, but the liquid system started to suffer from obvious capacity fading after 150 cycles. In contrast, the capacity retention rate of the AIO system was much better. The non-3D anode (Zn-foil-based AIO electrode) delivers inferior endurance to that of the 3D anode. The XRD patterns and SEM images of the two cathodes are shown in Fig. S10. For the NVO system at a current density of 10 A g^–1^, the liquid system first maintained a stable cycle of ∼500 times, but then a ‘cliff’ capacity decline occurred at the 530th cycle, which was associated with the severe H_2_ evolution phenomenon and the dissolution of zinc metal (inset of Fig. S11), as reflected in the CE shown in Fig. S11. In addition, because of the internal pressure increment, electrolyte leakage occurred on the corresponding button cell of the liquid system (Fig. S12). Because the AIO system can effectively inhibit hydrogen evolution, there was no obvious capacity decline even when the cycle number was up to 1000 (Fig. 4h), and the corresponding button battery was no electrolyte leakage.

To display the potential application of the AIO electrodes, we conducted the following experiments. We assembled a soft packing battery with an AIO electrode, and its first cycle CE reached ∼100% (Fig. S13a). Two AIO-based ZIBs were connected in series to power an LED bulb (rated voltage: 3 V). To simulate situations that may be encountered in actual applications, bending experiments (Fig. S13b and c; Video S1), piercing experiments (Video S2) and impact experiments (Video S3) were conducted. In all the above situations, the AIO ZIBs exhibited constant stability.

## CONCLUSION

In summary, an AIO electrode inheriting the advantages of the 3D zinc anode and gel electrolyte with almost no hydrogen evolution was prepared by two-step electroplating. In contrast to the point-to-surface contact between gel membranes and 3D zinc anodes in the past, the gel electrolyte here is tightly integrated with the Cu foam@Zn, providing more active sites and channels for redox reactions and fast ion transportation. As most water molecules in the gel electrolyte are constrained, hydrogen evolution is greatly suppressed. Therefore, the AIO electrode can effectively reduce side reactions, improve stability and obtain a relatively flat morphology. Consequently, compared with a 3D anode in routine liquid electrolyte, the AIO electrode exhibited a more stable CE (99.6%) under 20% depth of discharge. The stability of AIO electrode was confirmed using full batteries with NVO and *α*-MnO_2_ cathodes. In liquid electrolytes, zinc dissolution caused by strong gas evolution induced a sharp capacity decline; however, the capacity retention rate of the AIO system was as high as 85.4% (charging capacity), even after 1000 cycles. With this integrated AIO strategy, we hope to point out a way to combine modification methods and promote the development of next-generation Zn-based batteries.

## Supplementary Material

nwab177_Supplemental_Files
