# Supplementary material for: Integrated ‘all-in-one’ strategy to stabilize zinc anodes for high-performance zinc-ion batteries
Source: Natl Sci Rev. 2021 Sep 15;9(3):nwab177. doi: 10.1093/nsr/nwab177 (PMC8900688; doi:10.1093/nsr/nwab177)
Supplement: nwab177_Supplemental_Files [file nwab177_supplemental_files.zip › Supplementary_data.docx]

**Supporting Information for**

**Integrated “all-in-one” strategy to stabilize zinc anodes for**

**high performance Zn ion batteries**

*Canpeng Li^1,†^, Xuesong Xie^1,†^, Hui Liu^1^, Pinji Wang^1^, Canbin Deng^1^, Bingan Lu^2^, Jiang Zhou^1,3,*^, Shuquan Liang^1,3,*^*

^1^ School of Materials Science and Engineering, Central South University, Changsha, Hunan, 410083, China.

^2^ School of Physics and Electronics, Hunan University, Changsha 410082, P. R. China

^3^ Key Laboratory of Electronic Packaging and Advanced Functional Materials of Hunan Province, Changsha, Hunan, 410083, China.
*^*^* **Corresponding** **author.** E-mail: [zhou_jiang@csu.edu.cn](mailto:zhou_jiang@csu.edu.cn), [lsq@csu.edu.cn](mailto:lsq@csu.edu.cn)

^†^ Equally contributed to this work.

**Experimental Section**

**Preparation of raw materials.** ZnSO_4_·7H_2_O, MnSO_4_·H_2_O, Na_2_SO_4_, NH_4_VO_3_, H_2_C_2_O_4_·2H_2_O, boric acid, and sodium alginate were purchased from Sinopharm Chemical Reagent Co, Ltd. Palygorskite powder (800 mesh) was purchased from Kaiqi mineral processing plant. Zn foil (20 μm in thickness), Ti foil, Cu foam (99.9 wt%, 1.50 mm in thickness was purchased from Shanxi Lizhiyuan Battery Material Co., Ltd.

**Zn metal plating solution.** 30 g Na_2_SO_4_, 30 g ZnSO_4_·7H_2_O and 5 g boric acid were dissolved in 200 mL deionized water (DI) to form transparent solution under magnetic stirring.

**Gel electrolyte “plating” solution.** 25g sodium alginate and 40g palygorskite powder were mixed with 600 ml DI to form a homogeneous suspension under vacuum stirring.

**Preparation of Cu foam@Zn.** Cu foam@Zn was prepared according to our previous work with some modification. In details, Zn foil (5 cm x 7cm) as counter/reference electrode, Cu foam (1.8 cm x 2.5 cm) as working electrode. Before electroplating, Cu foam was washed by 1 M HCl solution to remove the oxides and cleaned with DI and ethanol. Subsequently, Zn was electroplated on Cu foam for 10 min under a constant current density of 40 mA cm^-2^. Finally, the products were cleaned with DI and ethanol. The amount of deposited zinc is 7.0 mA h cm^-2^ that we used in the long-term galvanostatic cycling performance of symmetrical cells. **Preparation of AIO electrode.** Cu foam@Zn as counter/reference electrode, Zn foil (2 cm x 6 cm) as working electrode. Gel electrolyte would combine with Cu foam@Zn under a constant current density of 10 mA cm^-2^ for 6 min. A Cu foam/Zn/gel electrolyte AIO electrode was obtained. Then the products were soaked in 2 M ZnSO_4_ (V-based cathode system) or 2 M ZnSO_4_ + 0.1 M MnSO_4_ (Mn-based system) to complete Zn ions crosslinking reaction of gel electrolyte. The thickness of gel electrolyte in the AIO electrode is about 1.0 mm.

**Preparation of NH_4_V_4_O_10_.** NH_4_V_4_O_10_ was prepared according to our previous work. In detail, 1.170 g NH_4_VO_3_ was dissolved in 80 °C deionized water to form a light yellow solution. Subsequently, 1.891 g H_2_C_2_O_4_·2H_2_O solid powders were added to the solution under magnetically stirring until it became black-green. The solution was transferred to a 50 mL autoclave and kept in an oven at 140 °C for 48h. The products were collected and washed repeatedly with de-ionized water after the sample was cooled to room temperature naturally. The final product was dried at 60 °C for 12h to obtain the NH_4_V_4_O_10_.

**Preparation of α-MnO2.** 0.00225 mol MnSO4·H2O is added to 15 ml deionized water and stirred it until a clear solution was obtained. Then, 15 ml 0.1 M KMnO4 aqueous solution was slowly added into the above solution. The mixture was stirred at room temperature for 1 h. The solution was then transferred to a Teflon-lined autoclave and heated at 160 ^o^C for 12 h. After cooling, the obtained material was collected by centrifugation, washed three times with deionized water, and dried in air oven at 60 ^o^C.

**Characterization.** Tafel plot, linear sweeping voltammograms (LSV), cyclic voltammetry (CV) curves, chronoamperometry, and electrochemical impedance spectra (EIS) were measured using an electrochemical workstation (MUL TI AUTOLAB M204). X-ray diffraction spectra were conducted by a Rigaku Mini Flex 600 diffractometer with a Cu K_α_-radiation (λ= 1.5418). Morphology SEM pictures were tested on a FESEM field emission scanning electron microscope (FEI Nova NanoSEM 230, 10 kV), and its corresponding energy dispersive X-ray spectrometer (EDS). FTIR was measured by the Fourier Transform Infrared Spectrometer (FTIR, Nicolet 6700). Galvanostatic charge/discharge cycling performances were carried out on a LANHE multichannel battery test system (CT2001A, China).

**Calculations:**

Ionic conductivities were tested by two blocking electrodes and calculated using the following equation:

$$\sigma=\frac{l}{R\cdot A}$$

where *R* represents the resistance according to EIS measurement, *l* represents the thickness of the membrane or gel electrolyte, and *A* is the area of the contact between blocking electrode and electrolyte.

The depth of discharge (DOD) of Zn anode was calculated according to the following equation:

$$DOD=\frac{It}{mM}\times100\%$$

where *I* (mA) is the applied current density, *t* (h) is the discharge time, *m* (g) is the mass of the Zn in Zn anode, *M* (mAh g^-1^) is the theoretical specific capacity of Zn (820 mA h g^-1^).

Float charge current: The batteries have been charged to full capacity at 1.8 V under constant current charging mode, then they are float charged constantly at 1.8 V for 120 h.

**Table S1**. Detailed data of float charge current

| Float current | Number of data points | | Sum  (mA) | Minimum (mA) | Average (mA) | Maximum (mA) |
| --- | --- | --- | --- | --- | --- | --- |
| Liquid | 589275 | 4580.79 | | 0 | 0.00777 | 0.3418 |
| AIO | 587945 | 2907.98 | | 0 | 0.00495 | 0.3196 |

**Supplementary figures and discussion**





Figure. S1 FT-IR spectra of gel electrolyte (mixed electrolyte) and pure sodium alginate.


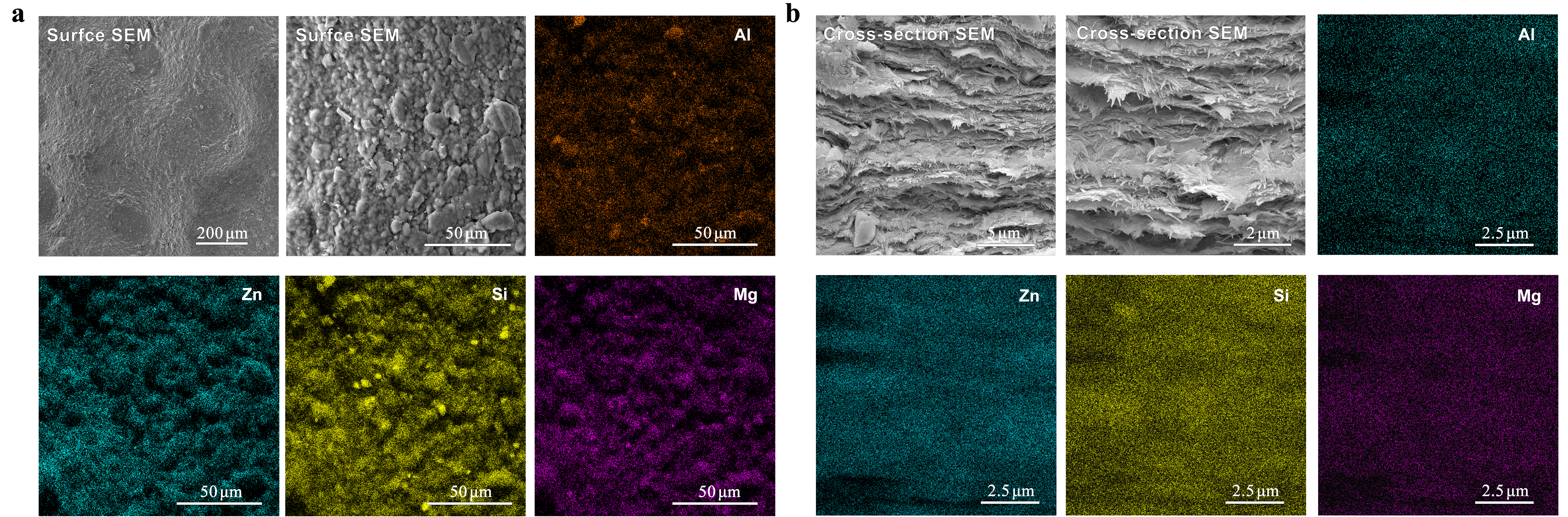


Figure. S2 The SEM images and its corresponding EDS mapping of gel membrane for the surface (a), and cross-sections (b). This result confirms the uniform distribution of palygorskite materials (MgAlSi_4_O_10_(OH)·4H_2_O) at the surface and body of gel membrane.


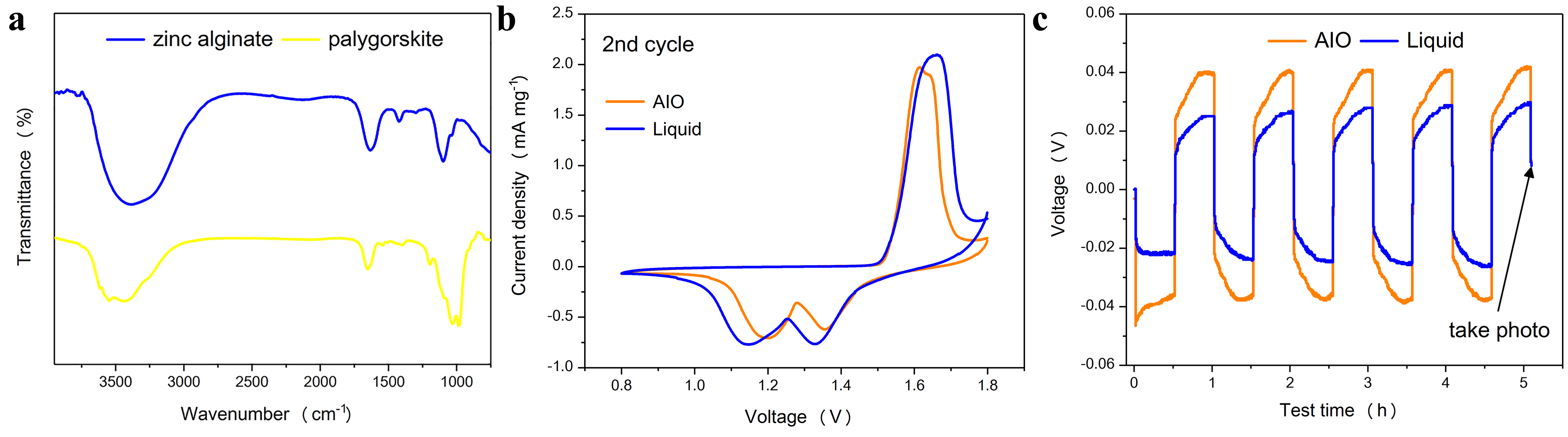


Figure. S3 (a) FT-IR spectra of zinc alginate and palygorskite. (b) The second cyclic voltammetry curve of Cu foam@Zn/α-MnO_2_ full cell. (c) The initial five detailed voltage profiles of symmetrical cells in transparent tanks.


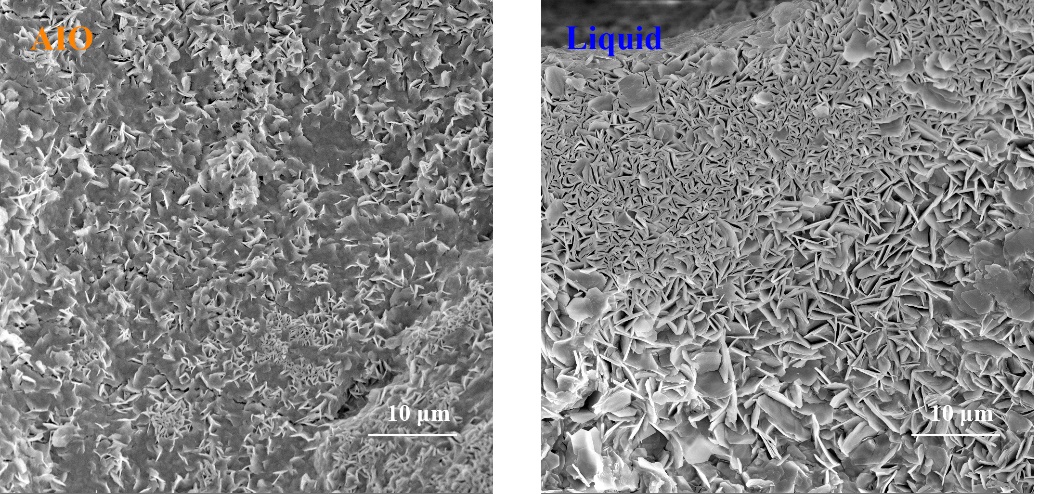


Figure. S4 The corresponding anode SEM images of cell with AIO electrode and Cu foam@Zn after 100 cycles in 2 M ZnSO_4_+0.1 M MnSO_4_.


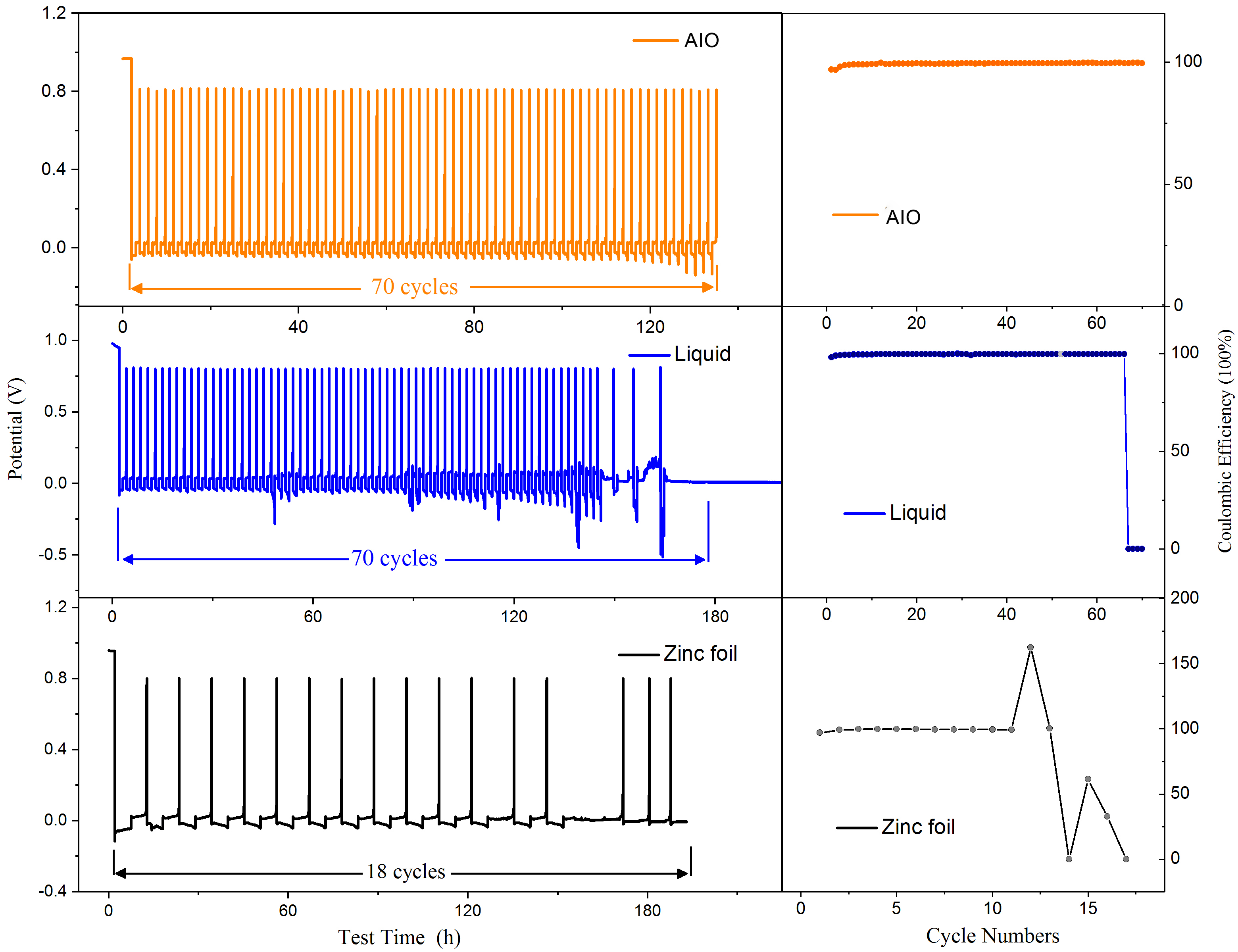


Figure. S5 Charge/discharge curves and Coulombic efficiency of Zn anode (Cu foam@Zn and Zinc foil)/Cu foil cell with 20% depth of discharge.


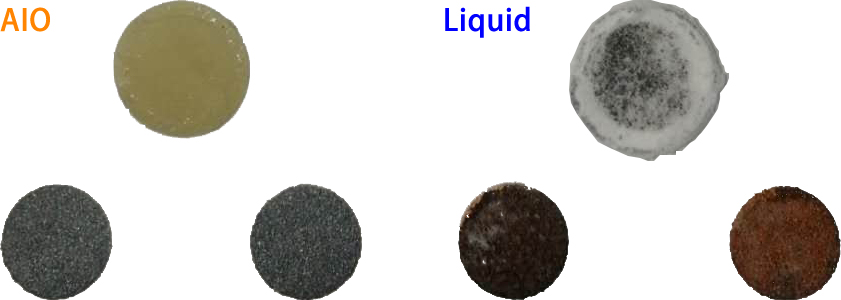


Figure. S6 Photos of symmetrical battery’s internal components after cycling.

**
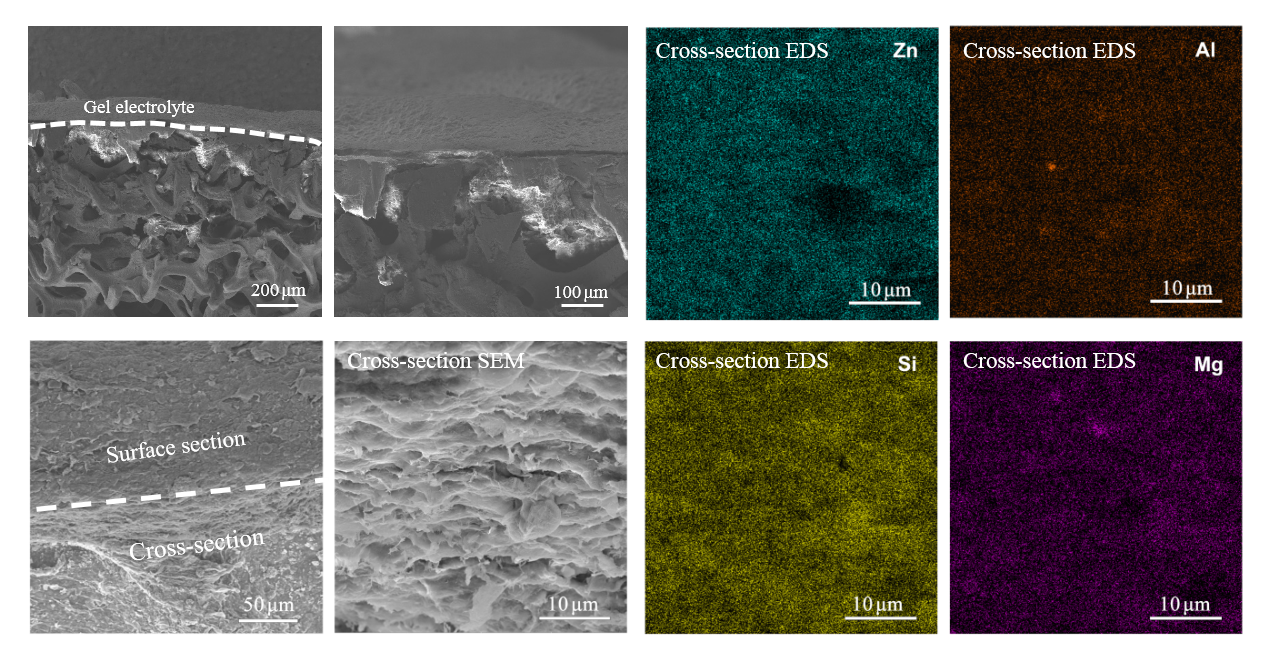
**

Figure. S7 The surface and cross-section SEM images of AIO after cycled in symmetric batteries, and its cross-section EDS mapping of gel electrolyte.


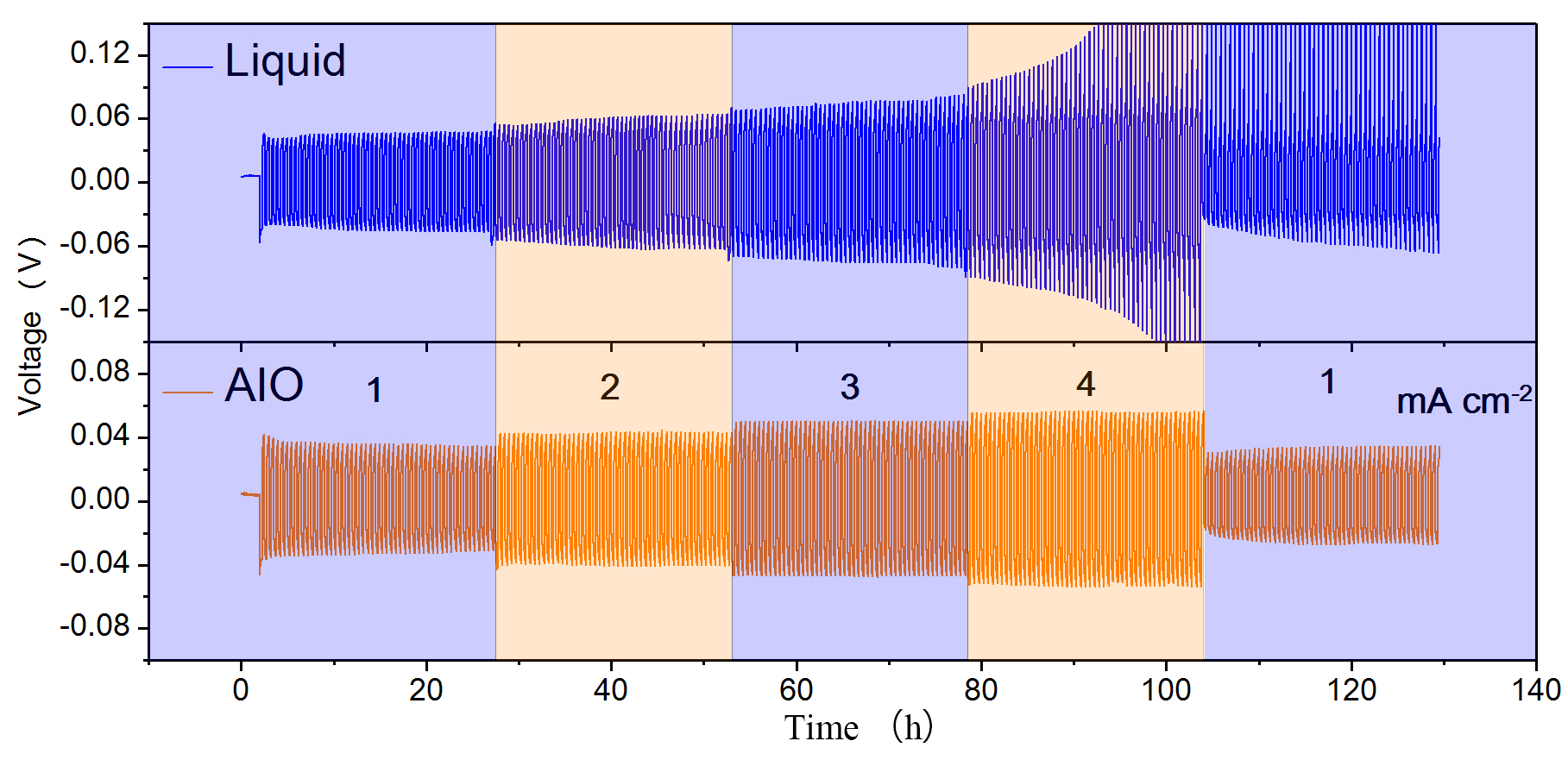


Figure. S8 Rate performance of symmetrical cells with AIO electrode and Cu foam@Zn.


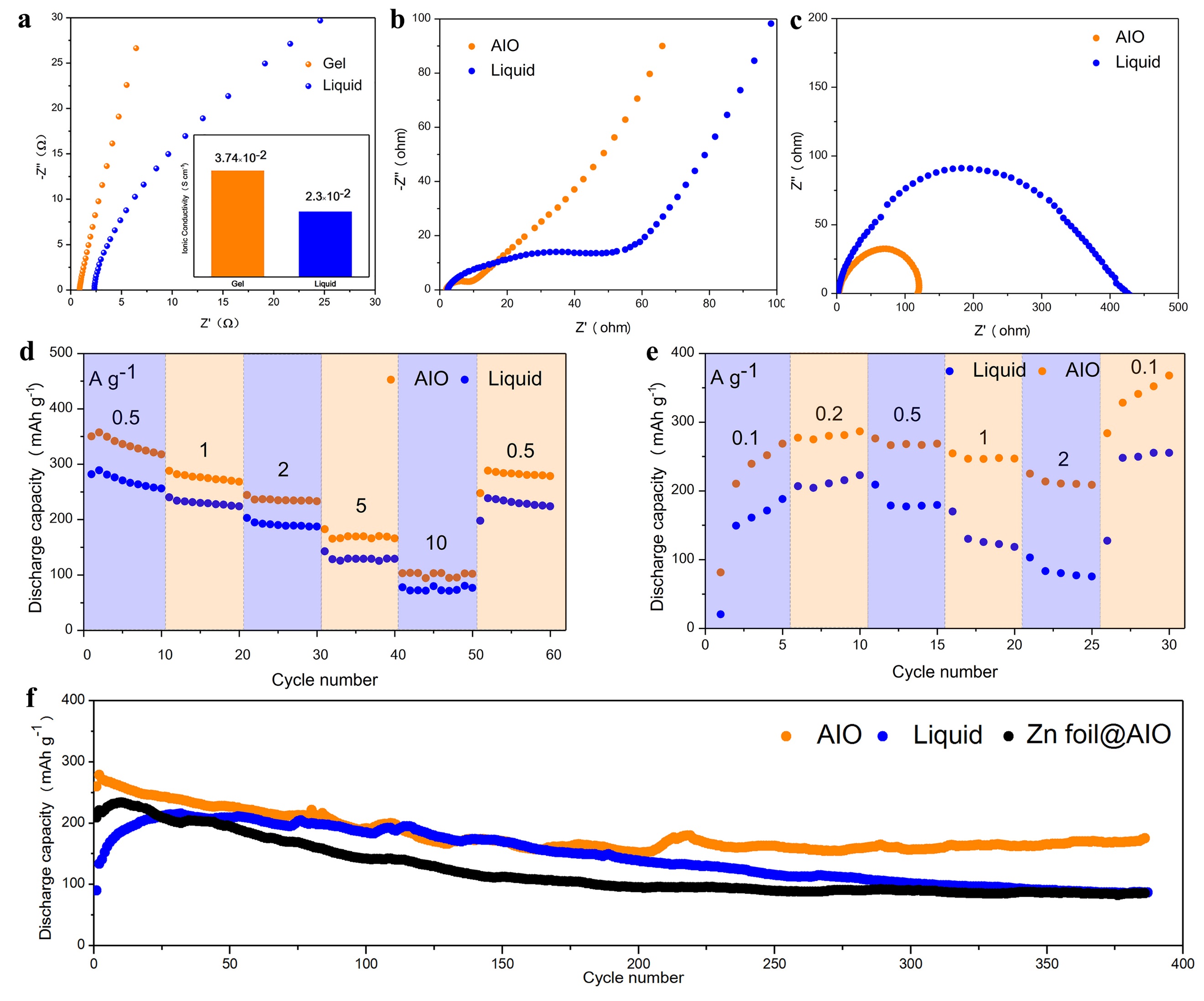


Figure. S9 (a) AC impedance spectra of the gel electrolyte and 2 M ZnSO_4_ aqueous electrolyte. (b) Nyquist plots of Cu foam@Zn/α-MnO_2_ battery. (c) Nyquist plots of symmetrical cells. Rate performance of (d) Cu foam@Zn/NH_4_V_4_O_10_ full cell and (e) Cu foam@Zn/α-MnO_2_ full cell. (f) Cycling performance of Cu foam@Zn/α-MnO_2_ full cells with AIO electrode, liquid electrolyte, and Zn foil-based AIO/α-MnO_2_ full cell (Zn foil@AIO) at a current density of 500 mA g^-1^.


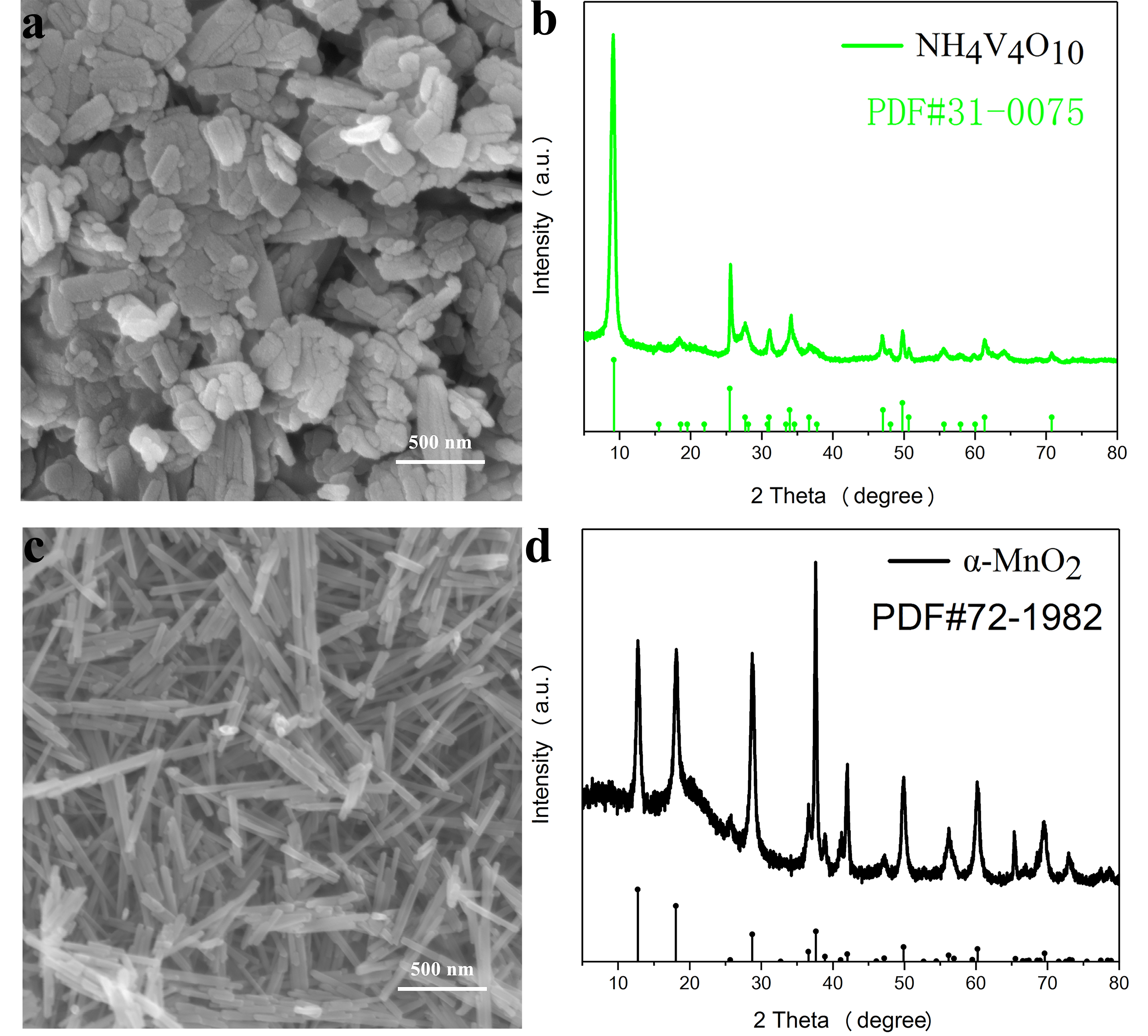


Figure. S10 (a) SEM image and (b) XRD pattern of NH_4_V_4_O_10_. (c) SEM image and (d) XRD pattern of α-MnO_2_.


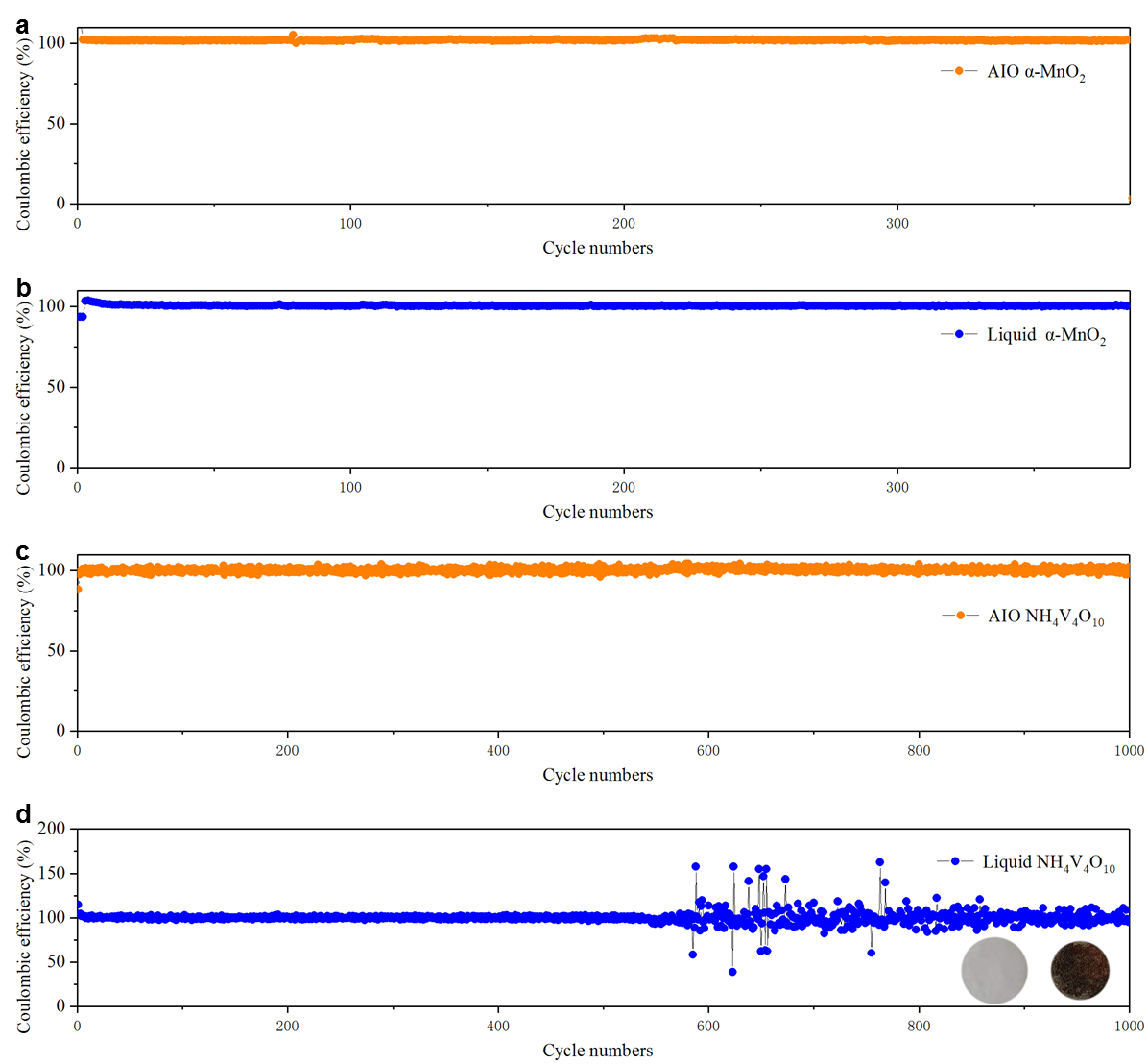


Figure. S11 Coulombic efficiency of (a) AIO electrode/α-MnO_2_ cell. (b) Cu foam@Zn/α-MnO_2_ cell. (c) AIO electrode/NH_4_V_4_O_10_ cell. (d) Cu foam@Zn/ NH_4_V_4_O_10_ cell.


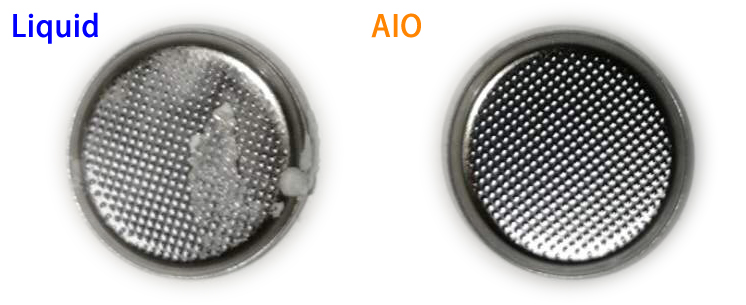


Figure. S12 Photo of full battery after cycling.


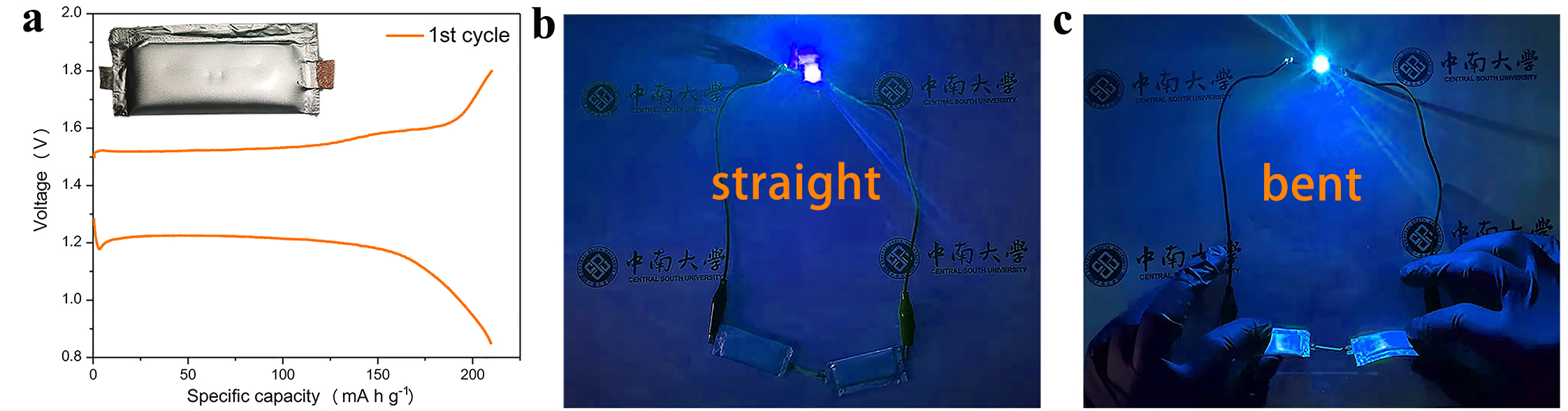


Figure. S13 (a) Photo of soft packing battery with AIO electrode and *α*-MnO_2_ cathode, and its 1st cycle performance. Soft packing battery with AIO electrode under (b) straight condition, (c) bending condition.
